# CaMKII Inhibition is a Novel Therapeutic Strategy to Prevent Diabetic Cardiomyopathy

**DOI:** 10.3389/fphar.2021.695401

**Published:** 2021-07-26

**Authors:** Christopher R. Veitch, Amelia S. Power, Jeffrey R. Erickson

**Affiliations:** Department of Physiology and HeartOtago, University of Otago, Dunedin, New Zealand

**Keywords:** CaMKII, diabetes, O-GlcNAc, oxidation, nitrosylation, arrhythmia, cardiomyopathy

## Abstract

Increasing prevalence of diabetes mellitus worldwide has pushed the complex disease state to the foreground of biomedical research, especially concerning its multifaceted impacts on the cardiovascular system. Current therapies for diabetic cardiomyopathy have had a positive impact, but with diabetic patients still suffering from a significantly greater burden of cardiac pathology compared to the general population, the need for novel therapeutic approaches is great. A new therapeutic target, calcium/calmodulin-dependent kinase II (CaMKII), has emerged as a potential treatment option for preventing cardiac dysfunction in the setting of diabetes. Within the last 10 years, new evidence has emerged describing the pathophysiological consequences of CaMKII activation in the diabetic heart, the mechanisms that underlie persistent CaMKII activation, and the protective effects of CaMKII inhibition to prevent diabetic cardiomyopathy. This review will examine recent evidence tying cardiac dysfunction in diabetes to CaMKII activation. It will then discuss the current understanding of the mechanisms by which CaMKII activity is enhanced during diabetes. Finally, it will examine the benefits of CaMKII inhibition to treat diabetic cardiomyopathy, including contractile dysfunction, heart failure with preserved ejection fraction, and arrhythmogenesis. We intend this review to serve as a critical examination of CaMKII inhibition as a therapeutic strategy, including potential drawbacks of this approach.

## Introduction

Diabetes Mellitus (DM) is a global health problem of rapidly increasing importance affecting millions worldwide, with this number only set to increase (Ogurtsova et al., 2017). DM is culpable for an increased susceptibility to cardiovascular disease (CVD) mortality among afflicted patients, this link being established by the landmark Framingham study (Garcia et al., 1974), which has been further corroborated by multitudes of other independent researchers (Kessler, 1971; Kannel and McGee, 1979; Gu et al., 1998). In order to lessen the significant impact of CVD-related morbidity within the diabetic population, novel molecular targets need to be investigated for their therapeutic benefit, and one such emerging target is the multifunctional enzyme Calcium/calmodulin (Ca^2+^/CaM) dependent protein kinase II (CaMKII).

Calcium/calmodulin (Ca^2+^/CaM) dependent protein kinase II (CaMKII) is an important nodal signalling molecule in the cardiovascular system, playing a number of roles in Ca^2+^ signal transduction pathways (for review, Hudmon and Schulman (2002) that are essential to normal cardiac function such as excitation contraction coupling (ECC). CaMKII activation contributes to tuning of the sinoatrial node (SAN) function (Vinogradova et al., 2000) as well as underlying the chronotropic response of the SAN to *β*-adrenergic stimulation (Wu et al., 2009). Chronic activation of cardiac CaMKII is associated with increased myocyte apoptosis (Zhu et al., 2003), fibrosis (Luo et al., 2013), hypertrophy (Ljubojevic-Holzer et al., 2020) and heart failure (Hoch et al., 1999), all of which are also observed in animal models and clinical presentation of diabetes [for review, Murtaza et al. (2019)]. CaMKII is also central in many arrhythmogenic processes (Erickson and Anderson, 2008), along with the heightened arrhythmia seen in diabetic cardiomyopathy (DCM) (Daniels et al., 2015). This review aims to compare emerging evidence as to the roles of different novel CaMKII activation pathways in the diabetic heart, and the pathological consequences of these modifications.

## Calcium/Calmodulin (Ca2+/CaM) Dependent Protein Kinase II Activation in Diabetes

**FIGURE 1:**
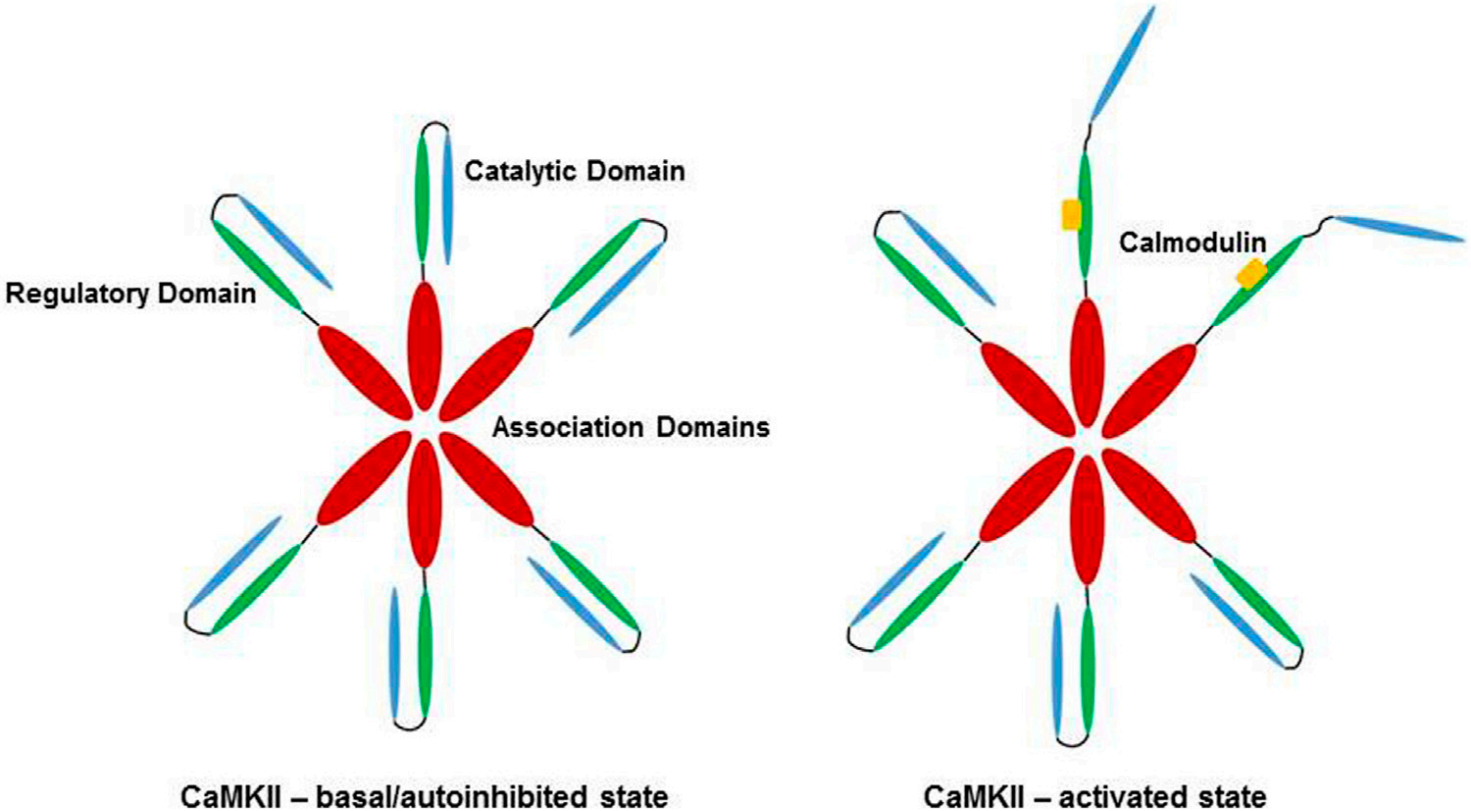
Activation of CaMKII by Ca^2+^/CaM. In low Ca^2+^/CaM concentrations, the catalytic domain (blue) is autoinhibited by the regulatory domain (green). Subunits of the dodecamer are held together by the association domains (red). High Ca^2+^/CaM concentrations (yellow) lead to binding of Ca^2+^/CaM to the regulatory domain, releasing the catalytic domain of CaMKII and allowing for enzymatic activity. Veitch 2021, original work.

CaMKII is a dodecameric holoenzyme, consisting of three distinct domains. The association domain allows for assembly of the various subunits into a dodecamer, the catalytic domain interacts with substrates and performs the kinase activity of the enzyme, and the regulatory domain interacts with the catalytic domain to sterically occlude substrate binding, resulting in autoinhibition of CaMKII at basal conditions (Rosenberg et al., 2005; Erickson, 2014). The regulatory domain also contains a calmodulin-binding region, and association with Ca^2+^/CaM disrupts the interaction between the regulatory and catalytic domains, activating the kinase and allowing CaMKII to phosphorylate targets. High or persistently elevated Ca^2+^/CaM concentrations result in autophosphorylation of the enzyme, allowing for subsequent Ca^2+^/CaM independent activity (Lai et al., 1986; Rosenberg et al., 2005). Activation of CaMKII is shown schematically in Figure 1.

**FIGURE 2:**
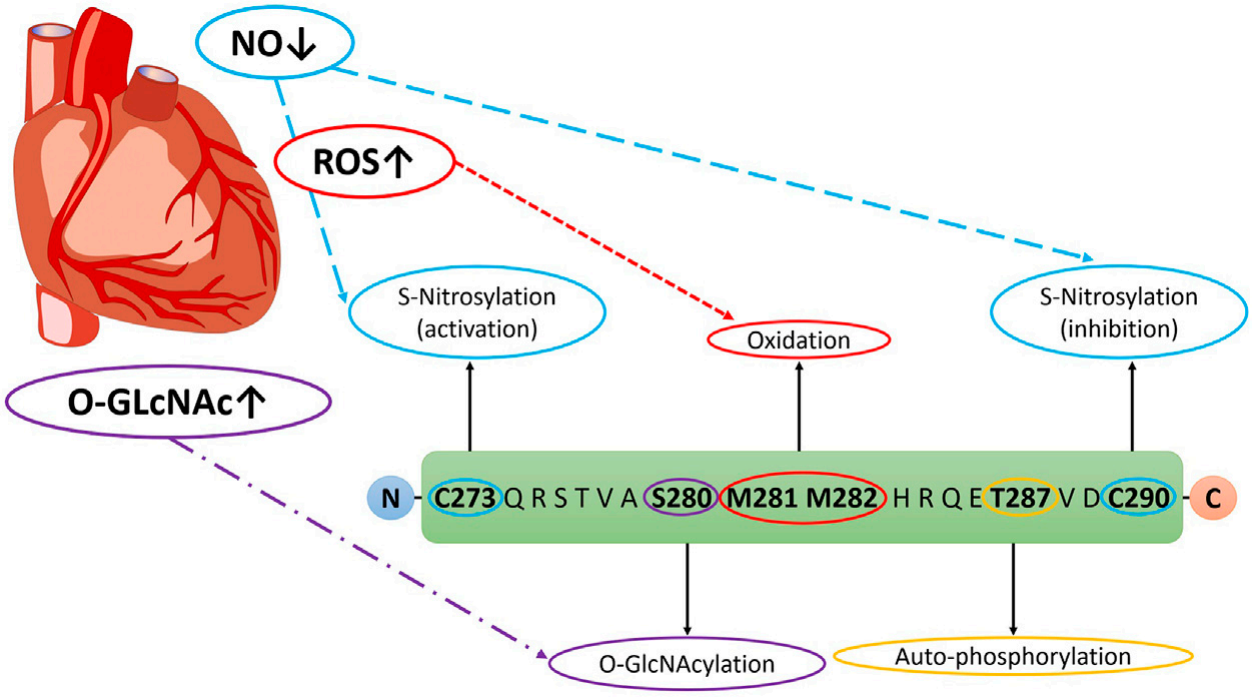
Post-translational modifications on CaMKII Regulatory Domain in DM. Veitch 2021, original work.

In addition to autophosphorylation, CaMKII can be chronically activated by a variety of post-translational modifications (PTMs) (Erickson, 2014), many of which take on increased prominence in the context of the diabetic milieu (Daniels et al., 2015). Indeed, elevated levels of blood glucose, increased calcium flux, enhanced production of reactive oxygen species (ROS) and aberrant nitric oxide signalling are well characterised in the setting of diabetes (Varga et al., 2015). Importantly, all of these cellular stresses are known to activate CaMKII. However, the contributions of each of these PTMs to the activation of CaMKII in the setting of DM has not been fully delineated. This section will aim to highlight the different modifications occurring to the enzyme in DM, along with summarising conflicting viewpoints on the contributions of various PTMs. The various PTMs occurring to the regulatory domain of CaMKII are highlighted in Figure 2.

### Oxidation in Diabetes

Oxidation of methionine residues 281/282 leads to Ca^2+^/CaM-independent autonomous activation of CaMKII akin to autophosphorylation (Erickson et al., 2008). Levels of pro-oxidants and oxidised proteins are heightened in both the plasma and pancreas of human and animal models, from the various effects of hyperglycemia on inflammatory processes and alteration of anti-oxidant enzymes [for review, Rehman and Akash (2017)].Mitochondrial ROS production in the myocardium is known to be increased in both T1 (Pham et al., 2014) and T2DM (Boudina et al., 2007). Oxidised CaMKII (ox-CaMKII) has been implicated as an arrhythmogenic substrate in diabetes and may play an integral role in the greater mortality rates seen in diabetic patients and animal models (Luo et al., 2013; Mesubi et al., 2020). For example, ox-CaMKII increases the risk of mortality following a myocardial infarction in diabetic and non-diabetic mouse models (Erickson et al., 2008; Luo et al., 2013).

Diabetes is strongly associated with arrhythmia in human patients (Movahed et al., 2005) and ox-CaMKII may underlie this increased risk of arrhythmogenesis. Ox-CaMKII augments the late Na^+^ current (*I*_Na_) in isolated ventricular cardiomyocytes from WT mice and rabbits, leading to accumulation of cytosolic Na^+^ and increased Ca^2+^ entry *via* the sodium/calcium exchanger (NCX) (Wagner et al., 2011). This ultimately leads to Ca^2+^ overload, contractile dysfunction and hypercontracture, along with Ca^2+^ sparks and SR Ca^2+^ leak (Wagner et al., 2011). Blocking CaMKII in mouse ventricular myocytes with inhibitor KN-93 was unable to affect H_2_O_2_-induced increases in Ca^2+^ sparks, suggesting that oxidative stress resulted in autonomous activation of the enzyme, preventing the inhibitory action of KN-93, which can only block CaMKII activity if the enzyme has not been autonomously activated (Buard et al., 2010). Investigators have also suggested that CaMKII acts as a sensor for ROS, translating heightened oxidative stress into enhanced susceptibility to atrial fibrillation (AF) showing a resistance to AF induction in a mouse model missing oxidation sites on CaMKIIδ (MM-VV) (Purohit et al., 2013). This group further identified significantly increased levels of ox-CaMKII in human AF patients, implicating ox-CaMKII in the pathogenesis of human AF (Purohit et al., 2013). Interestingly, human AF patients treated with ACE inhibitors or angiotensin receptor blockers did not show similarly elevated levels of ox-CaMKII as untreated patients (Purohit et al., 2013) consistent with prior observations connecting elevated angiotensin II and aldosterone to the generation of excess ox-CaMKII (Erickson et al., 2008; He et al., 2011). Murine models of Duchenne muscular dystrophy show significantly increased ox-CaMKII, which may be linked to early stages of dystrophic cardiomyopathy (Wang et al., 2018). Ox-CaMKII may also mediate the cardiotoxic/arrhythmic side-effects of cardiac glycosides, with excess ROS generated by NOX2 oxidizing CaMKII acting on RyR2 to generate proarrhythmic Ca^2+^ waves (Ho et al., 2014). Taken together, these studies suggest that therapies focused on preventing CaMKII oxidation could alleviate arrhythmogenesis in the diabetic heart. The interactions of ox-CaMKII with various molecular targets associated with arrhythmia in both diabetes and other disease models suggest a pivotal role of ox-CaMKII in the generation of arrhythmogenic events. As oxidative stress is known to be heightened in diabetes, this modification could provide a useful target to alleviate the increased risk of arrythmia in diabetic patients.

### O-GlcNAcylation in Diabetes

O-GlcNAcylation is the PTM of a protein *via* addition of an O-linked *β*-N-acetylglucosamine (O-GlcNAc) residue onto proteins at serine or threonine residues. This modification is influenced by two enzymes; O-GlcNAc transferase (OGT) and O-GlcNAcase (OGA), which catalyze the addition and removal of O-GlcNAc residues respectively. This modification has garnered increasing interest due to its involvement in pathological processes associated with DM, in which the balance of signaling between phosphorylation and O-GlcNAcylation is disrupted (Butkinaree et al., 2010). The substrate for O-GlcNAcylation, UDP-GlcNAc, is regulated through the hexosamine biosynthesis pathway (HBP), which shuttles intracellular glucose through the pathway to produce UDP-GlcNAc for O-GlcNAcylation. In DM, glucose may be favoured to be metabolised through alternative pathways such as the HBP due to impairments in insulin signaling, leading to elevated concentrations of UDP-GlcNAc in diabetic mice (Clark et al., 2003). This increase in UDP-GlcNAc production causes an elevation in O-GlcNAc modifications on proteins, as has been observed in left ventricular and right atrial appendage samples from diabetic patients (Prakoso et al., 2021).

CaMKII can be O-GlcNAcylated at the S279 residue, causing autonomous activation in a similar manner to autophosphorylation and oxidation (Erickson et al., 2013). O-GlcNAcylated CaMKII is significantly increased in the hearts of diabetic rats and human diabetic patients (Erickson et al., 2013; Daniels et al., 2018). This increase in O-GlcNacylated CaMKII underpins a number of mechanisms that promote cardiac arrhythmia. Hyperglycemia-induced activation of CaMKII occurs *via* O-GlcNAc modification and induces spontaneous Ca^2+^ leak in isolated rat cardiomyocytes, premature ventricular contractions in *ex vivo* rat hearts, and increased arrhythmia susceptibility in diabetic rats during a caffeine stress test (Erickson et al., 2013). O-GlcNAc-CaMKII also modulates K^+^ channels, affecting both transient outward and rectifier K^+^ current in rodent myocytes, leading to increased arrhythmia susceptibility (Hegyi et al., 2019).

Clark, McDonough (Clark et al., 2003) showed that increased O-GlcNAcylation in cardiomyocytes results in abnormalities in Ca^2+^ flux, which can be remedied by inhibiting O-GlcNAcylation. Interestingly, this amelioration of Ca^2+^ flux occurs despite ongoing hyperglycaemia, suggesting that O-GlcNAcylation and not hyperglycaemia is causing these overt disruptions in Ca^2+^ transport (Clark et al., 2003). In a Zucker diabetic fatty (ZDF) rat model of type II diabetes, Fülöp et al. (2007) found these animals exhibited impaired cardiac relaxation, depressed peak Ca^2+^ transient levels and slower rates of Ca^2+^ decay, which were associated with increased levels of O-GlcNAcylation. In addition to altering Ca^2+^ flux, excessive O-GlcNAcylation can produce contractile dysfunction in diabetic cardiomyopathy, *via* affecting Ca^2+^ sensitivity of the myofilaments (Ramirez-Correa et al., 2015). Although these studies do not directly implicate O-GlcNAc-CaMKII in diabetes, the studied abnormalities are well characterised to be directly affected by CaMKII (Ca^2+^ fluxand cardiac contraction) so it is not far-fetched to suggest that CaMKII may be playing an intermediary role in these O-GlcNAc mediated dysfunctions. However, Fülöp et al. (2007) only observed increased O-GlcNAc levels in high molecular weight proteins (≥205 kDa), much greater in weight than CaMKII (∼50–60 kDa). Considering the data of Fülöp et al. (2007) it would be prudent to investigate the interplay between CaMKII and high weight molecular proteins (such as ion channels) that may be contributing to arrhythmogenesis in DM.

Many of the cardiac pathologies influenced by O-GlcNAc-CaMKII seem to be alleviated by inhibition of either O-GlcNAcylation generally, or a more specific targeting of O-GlcNAc-modified CaMKII. Erickson et al. (2013) showed that both a direct genetic target of the CaMKII O-GlcNAc site (S279) was able to completely prevent glucose-induced CaMKII autonomous activation, along with acute treatment with DON (an inhibitor of the HBP) was able to prevent O-GlcNAc modification of CaMKII. DON treatment was able to reduce glucose-induced arrhythmia incidence and score in an *in vivo* diabetic rat model to a similar level as KN-93, showing the importance of both O-GlcNAc modification and CaMKII activation in diabetic arrhythmogenesis (Erickson et al., 2013). The cellular arrhythmia observed by Hegyi et al. (2019) in diabetic mouse cardiomyocytes was prevented by either acute inhibition of O-GlcNAcylation or genetic inhibition of either CaMKII or the O-GlcNAcylation site on CaMKII. Ramirez-Correa et al. (2015) found that removal of O-GlcNAcylation from myofilaments was able to restore myofilament function, showing that a more targeted approach of O-GlcNAc-CaMKII may prove beneficial in diabetic cardiomyopathy. By using a more targeted approach, a specific pathology (such as impaired myofilament Ca^2+^ sensitivity) may be effectively alleviated whilst also minimizing the potential of unintended side effects, given the physiological importance of O-GlcNAcylation in normal cellular function [for review, Butkinaree et al. (2010)].

### Calcium/Calmodulin (Ca2+/CaM) Dependent Protein Kinase II Oxidation and O-GlcNAcylation as Therapeutic Targets in the Diabetic Heart

Despite evidence showing activation and subsequent arrhythmia resultant from both O-GlcNAcylated and oxidised CaMKII, the relative contributions of these mechanisms and the efficacy of targeting them as a therapeutic approach remains unclear. In this section, we will present experimental evidence for the potential benefits of targeting either O-GlcNAcylated or oxidised CaMKII as an approach to prevent cardiac pathology in the context of DM.

Considering the extensive role ox-CaMKII plays in cardiac pathologies and the increased prevalence of oxidative stress in the diabetic heart, ox-CaMKII presents a tantalizing target to alleviate the morbidity resultant from oxidative stress in the diabetic milieu. Indeed, blocking ox-CaMKII preserved the physiological fight/flight response whilst eliminating the increased risk of death from myocardial infarction in diabetic mice (Luo et al., 2013), highlighting the potential therapeutic benefit of blocking ox-CaMKII in a clinical setting. In a whole mouse model lacking functional NADPH oxidase or with CaMKII inhibition, mice were highly resistant to sinus node dysfunction induced by angiotensin II infusion, suggesting that CaMKII plays an important role in this pathway (Swaminathan et al., 2011) and further demonstrating the therapeutic benefit of a targeted approach focused on ox-CaMKII in DM.

Sommese et al. (2016) observed increased spontaneous Ca^2+^ release events associated with CaMKII-mediated phosphorylation of RyR2 in cardiomyocytes isolated from mice fed a fructose-rich diet (FRD) compared to control animals. Furthermore, ROS production and expression of ox-CaMKII were significantly increased in FRD animals compared to control animals (Sommese et al., 2016). Treating FRD mice with an intracellular ROS scavenger or inhibition of CaMKII ablated this increase in spontaneous Ca^2+^ events and CaMKII phosphorylation of RyR2, leading the authors to conclude that ox-CaMKII is the main driver of arrhythmogenesis in this model, with any other activation of CaMKII (O-GlcNAcylation/nitrosylation) playing a minor role. A more recent study examined the differential effects of oxidation and O-GlcNAcylation on AF, concluding that the loss of the oxidation site on CaMKII (M281/282) was sufficient to ablate AF susceptibility in mouse models of both type 1 and type 2 DM (Mesubi et al., 2020). Unexpectedly, the authors found that loss of the O-GlcNAcylation site on CaMKII (S280) was insufficient to prevent AF in both their type 1 and type 2 DM mouse models. Acute inhibition of O-GlcNAcylation by pretreating mice with DON was able to protect against AF in T1DM, but not T2DM mice (Mesubi et al., 2020). The authors suggest that oxidation of CaMKII is the main driver of diabetic arrhythmogenesis, as opposed to a minor role of O-GlcNAcylation, particularly in T2DM. Considering the proximity of both the O-GlcNAcylation and oxidation sites on CaMKII, the authors made note that these modifications could be influencing each other, affecting the resultant arrhythmogenesis from either modification. The suggestion of interplay between O-GlcNAcylation and autophosphorylation of CaMKII (Erickson et al., 2013) provides precedent for this argument, which could be an interesting research avenue in the setting of DM.

Another body of published evidence instead supports O-GlcNAcylation as having a dominant role in diabetes-induced cardiac pathology. Lu et al. (2020) showed that ROS production resultant from high glucose is a downstream consequence of O-GlcNAcylation and CaMKII activation, as they observed a complete ablation of high glucose induced ROS production and Ca^2+^ sparks when cardiomyocytes were treated with CaMKII or HBP inhibitors (Fülöp et al., 2007). In direct contrast to Mesubi et al. (2020), they also showed that mutating the O-GlcNAcylation site on CaMKII is sufficient to prevent SR Ca^2+^ leak, whilst mutation of the oxidation site on CaMKII or inhibition of ROS production is insufficient to prevent spontaneous Ca^2+^ leak from the SR. One potential explanation for this discrepancy is that Mesubi et al. (2020) studied a diabetic model partially induced by streptozotocin (STZ) treatment (Hegyi et al., 2019), whilst Lu et al. (2020) examined a cardiomyocyte model incubated with high glucose and no STZ treatment for induction of diabetes (Fülöp et al., 2007). Recently, Hegyi et al. (2021a), Hegyi et al. (2021b) found that mutation of the CaMKII O-GlcNAcylation site significantly ablated the occurrence of arrhythmia in a diabetic hyperglycaemic mouse model, whilst preventing CaMKII oxidation had no effect during acute hyperglycaemia and only a minor effect in a mouse model of chronic diabetes.

Another study by Hegyi et al. (2020) supports the theory that O-GlcNAcylation and oxidation may be influencing each other, but it also emphasizes a more prominent role for O-GlcNAcylation of CaMKII in the context of diabetes. O-GlcNAcylated CaMKII was observed to alter K^+^ channels currents, such as a reduction in I_to_ recovery and I_K1_ amplitude, which may contribute to arrhythmia in diabetes. I_to_ amplitude was also seen to be reduced in acute hyperglycaemia, although this effect was not dependent on O-GlcNAc-CaMKII, instead seeming to be regulated *via* a NOX2-ROS-PKC pathway.

The possibility also exists that O-GlcNAcylation may be affecting the diabetic heart in a CaMKII-independent manner. A recent study by Umapathi et al. (2021) showed that overexpression of OGT was able to induce cardiomyopathy and increases in Ca^2+^ spark frequency post-trans-aortic constriction in a mouse model. OGT-overexpressing mice also exhibited dramatically increased mortality rates compared with a control group; however, these effects were not alleviated by mutation of the O-GlcNAcylation site on CaMKII, S280 (Umapathi et al., 2021). Perhaps an effect unique of the diabetic state is influencing CaMKII activation by O-GlcNAcylation in the other discussed studies, as opposed to O-GlcNAcylation in a non-diabetic model. It remains an open question as to whether O-GlcNAcylation in diabetes is influencing cardiac mortality in a CaMKII-independent manner.

Rather than being solely regulated by either O-GlcNAcylation or oxidation, arrhythmogenesis in diabetes may be regulated by both modifications interacting with each other at different stages of the disease process. The complex nature of DM makes it seemingly improbable that a pathological process is regulated by one factor, instead the answer may lie within crosstalk of these two modifications, as well as others. Further targeted research to fully elucidate the mechanism(s) by which the diabetic milieu activates CaMKII and contributes to cardiac pathology is needed.

### S-Nitrosylation in Diabetes

S-nitrosylation involves the PTM of protein thiols by nitric oxide (NO) and has recently emerged as a key regulator of cardiac signalling. NO is typically produced in the myocardium by various isoforms of NO synthase (NOS) and regulates a number of cardiac physiological processes [for review, Murphy et al. (2014)]. For instance, the interplay between NO and CaMKII is integral to mechanochemotransduction within cardiomyocytes (Jian et al., 2014). Regulation of NO production is impaired in diabetes and has been suggested to underlie the various vascular pathologies present in DM (Knapp et al., 2019). Interestingly, activation of the HBP impairs insulin-mediated NOS activity (Federici et al., 2002), suggesting a role of O-GlcNAcylation in diabetes mediated NOS impairment.

NO provides a novel activation mechanism for CaMKII in cardiac tissue by directly adding an S-nitrosylation modification to the protein (Gutierrez et al., 2013). NO donors were able to increase Ca^2+^ spark frequency in cardiomyocytes, with this effect almost completely ablated by inhibiting CaMKII (Gutierrez et al., 2013). Erickson et al. (2015) provided further insight into interactions between NO and CaMKII, showing a novel dual-regulation of CaMKII by NO. Nitrosylation of CaMKII at C290 resulted in autonomous activation of the enzyme, whilst nitrosylation at C273 inhibits CaMKII activity. The authors suggested that timing of Ca^2+^ release events could be a critical factor in determining whether CaMKII is activated or inhibited, due to Ca^2+^/CaM dependence of some NOS variants for NO production. Considering the well-studied changes in Ca^2+^ handling in DM, it would be an intriguing topic to investigate how DM influences CaMKII activation/inhibition by NO.

Enzymatic “uncoupling” of eNOS leads to a reduction in NO production and an increase in superoxide production. A reduction of BH_4_ levels, a cofactor essential for eNOS function, occurs in diabetes, which is associated with increased oxidative stress and reduced NO production (Alp et al., 2003). By increasing BH_4_ levels, oxidative stress is reduced and the loss of NO production is ameliorated (Alp et al., 2003). Along with a reduction in BH_4_ levels, increased O-GlcNAcylation in diabetes is correlated with reduced Akt phosphorylation (Luo et al., 2008) which is responsible for eNOS activation (Du et al., 2001). Along with this, O-GlcNAcylation of eNOS is increased, which may result in decreased eNOS activity (Du et al., 2001).

Despite the myriad of studies showing reductions in NO production in diabetes, the focus of much of this research is on vascular rather than cardiac dysfunction. Considering the results of Erickson et al. (2015) and Gutierrez et al. (2013) concerning the dual-regulation of CaMKII by NO, it could be reasoned that this regulation is altered in diabetes due to disruptions in NO production, potentially contributing to cardiac dysfunction in DM *via* pathological changes in CaMKII activation. The involvement of nitrosylated-CaMKII in both inotropy and generation of spontaneous Ca^2+^ sparks and waves have been shown in non-diabetic models (Gutierrez et al., 2013; Curran et al., 2014; Pereira et al., 2017). Both of these phenomena have been well characterised to be upset in the diabetic milieu, however no research as of yet has investigated the role of nitrosylated-CaMKII in these events in DM.

## Arrhythmogenic Targets of Calcium/Calmodulin (Ca2+/CaM) Dependent Protein Kinase II

**FIGURE 3:**
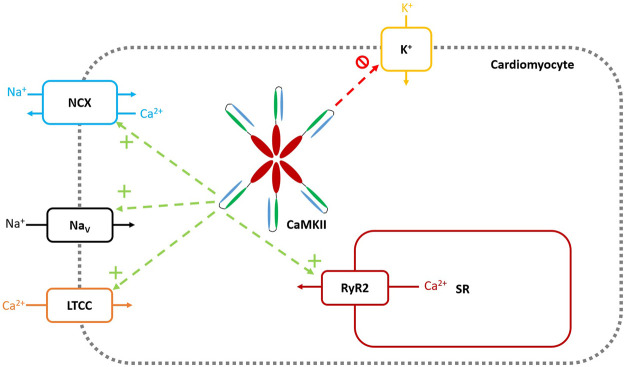
Molecular Targets of CaMKII. Green arrows and + symbols represent CaMKII increasing functional activity of the target channel through either upregulation of channel numbers, or direct augmentation of channel activity. Red arrows and symbols represent CaMKII decreasing functional activity of the target channel. Veitch 2021, original work.

Diabetes has long been recognized as an independent risk factor for arrhythmogenesis (Benjamin et al., 1994) and the downstream arrhythmogenic effects of CaMKII following post-translational modification in diabetes have been discussed above. Mechanistically, CaMKII may lead to proarrhythmic consequences *via* early or delayed after depolarisations (EADs and DADs), which lead to triggered activity and subsequent arrhythmia, such as atrial fibrillation [for review, Mustroph et al. (2017)]. EADs involve an early, secondary depolarisation prior to repolarization of the action potential, resultant from reopening of L-type Ca^2+^ channels (LTCC) (Dobrev and Wehrens, 2017). DADs occur following a full repolarization of the action potential, resultant from spontaneous Ca^2+^ release from the SR. CaMKII affects the development of arrhythmia *via* a plethora of different molecular targets, some of which will be discussed below. These molecular targets are summarised in Figure 3.

### Na^+^ Channels

Voltage gated Na^+^ channels (Na_V_) play an integral role in normal cardiac physiology, allowing for rapid Na^+^ entry into cardiomyocytes, resulting in the upstroke of the cardiac action potential, followed by fast inactivation allowing for repolarization (for review Marban et al. (1998)]. Alterations in this Na^+^ current through Na_V_ channel modification can have arrhythmogenic consequences, such as increasing the late Na^+^ current (I_Na_) or altering channel expression, leading to arrhythmia (Yu et al., 2018).

Oxidised CaMKII contributes to heightened late I_Na_ following activation of the renin-angiotensin system, resulting in generation of early after depolarisations (EADs) leading to increased propensity for cellular arrhythmia in isolated rabbit ventricular myocytes (Zhao et al., 2011). In a similar rabbit ventricular myocyte model, Ma et al. (2012) showed that inhibition of both PKC and CaMKII was able to prevent heightened late I_Na_ following Ca^2+^ overload, suggesting that multiple kinases may play a role in arrhythmias associated with late I_Na_. Howard et al. (2018) showed that this heightened late I_Na_ occurs in other disease states such as I/R injury in a mouse Langendorff model, proposing that CaMKII is directly hyperphosphorylating Na_V_1.5 leading to spontaneous arrhythmogenic events in I/R injury. Lebek et al. (2020) was able to expand upon these previous animal studies, showing increased phosphorylation of Na_V_1.5 in atrial myocytes from human patients with sleep disordered breathing. Atrial myocytes from these patients exhibited heightened late I_Na_, which was reversed by acute inhibition of CaMKII (Lebek et al., 2020). Late I_Na_ is heightened in diabetes, contributing to the arrhythmogenic substrate (Lu et al., 2013; Jin et al., 2019), which may be another pathway by which CaMKII influences arrhythmogenesis in DM. In an obese mouse model, ablation of the modification site by CaMKII on cardiac Na_V_1.5 was able to both reduce arrhythmogenesis and alleviate cardiac remodeling (Dewal et al., 2021). To the best of our knowledge, no study to date has examined the role of CaMKII in modulating cardiac Na_V_ in DM. These studies highlight the importance of Na_V_ in both normal physiological function and the importance of dysregulation of these channels in disease states resultant from modulation by CaMKII.

### Cardiac Ryanodine Receptor

The cardiac ryanodine receptor (RyR2) is integrally involved in cardiac ECC *via* the process of Ca^2+^ induced Ca^2+^ release (CICR). Depolarisation of the cardiomyocyte leads to Ca^2+^ influx *via* LTCC. This initial Ca^2+^ influx triggers RyR2 on the sarcoplasmic reticulum (SR) membrane to release Ca^2+^ stored within the SR, providing enough Ca^2+^ for cardiac contraction [for review, Wehrens and Marks (2003)]. CaMKII regulates RyR2 *via* phosphorylation (Wehrens et al., 2004), which may play an integral role in arrhythmogenesis during various disease states.

CaMKII phosphorylation of RyR2 increases Ca^2+^ leak from the SR, resulting in an increased propensity for arrhythmogenic events. CaMKII-mediated RyR2 Ca^2+^ leak is involved in arrhythmogenesis in a large variety of pathological states, including Duchenne muscular dystrophy (Ather et al., 2013), chronic *β*-adrenergic stimulation (Grimm et al., 2015), atrial fibrillation (Chelu et al., 2009) and arrhythmias resultant from cardiac glycoside treatment (Gonano and Petroff, 2014). This CaMKII-mediated SR Ca^2+^ leak may even play a role in apoptotic pathways, with this effect heightened during situations of hyperglycaemia (Federico et al., 2017). In T2DM rat hearts, CaMKII-mediated SR hyperphosphorylation enhances SR Ca^2+^ leak, increasing the propensity for DADs, and thus arrhythmia (Popescu et al., 2019).

A recent mathematical modelling study shows a potential relationship between Na_V_, RyR2 and CaMKII. CaMKII-dependent increases in late I_Na_ lead to increased intracellular Ca^2+^ *via* reverse-mode Na^+^/Ca^2+^ exchanger (NCX) activity, resulting in further enhanced CaMKII activity and an increased RyR2 open probability (Onal et al., 2017). This study shows the potential for various arrhythmogenic pathways/targets to synergise with each other in pathological situations, leading to positive feedback loops and heightening the risk for an arrhythmogenic event. Voigt et al. (2012) showed evidence of heightened Ca^2+^ spark frequency, increased opening probability of RyR2 and greater I_NCX_ in human AF myocytes. Inhibiting CaMKII was able to normalise the increased SR Ca^2+^ leak and reduce the opening probability of RyR2 (Voigt et al., 2012). As increased cytosolic Ca^2+^ activates forward-mode NCX, the authors suggest that the interplay between CaMKII, RyR2 and NCX results in the heightened Ca^2+^ spark frequency and DaDs in human AF myocytes.

### Na^+^/Ca^2+^ Exchanger

Along with its interplay with RyR2 in arrhythmogenesis, CaMKII may play a role in directly or indirectly facilitating the arrhythmogenic transient inward current (I_ti_)*.* Inhibition of CaMKII was able to prevent an NCX mediated I_ti_ in isolated rabbit ventricular myocytes, which may help prevent Ca^2+^ overload induced arrhythmia (Wu et al., 1999a). In an STZ-induced DM rat model, NCX protein expression is increased in atrial tissue, which was ameliorated by antioxidant treatment (Yang et al., 2020). This antioxidant treatment also alleviated the raised levels of phosphorylated CaMKII seen in this diabetic model. The authors concluded from these findings that CaMKII may be involved in regulation of NCX expression in diabetes, which could provide another pathway by which CaMKII increases arrhythmogenesis in diabetic patients. A recent study by Al Kury et al. (2021) in a similar STZ-induced DM rat model identified increased Ca^2+^ transient amplitudes in diabetic ventricular myocytes. This was accompanied with a significant impairment of NCX current amplitude in the diabetic cells. Increased amplitudes of Ca^2+^ transients may lead to the increased arrhythmia seen in both diabetic animal models and human patients.

### L-Type Ca^2+^ Channels

LTCC plays a crucial role in cardiac ECC, allowing influx of Ca^2+^ into the cardiomyocyte following membrane depolarisation. CaMKII regulates sino-atrial node pacemaker cells by modulating LTCC. Inhibition of CaMKII reduced LTCC current by 50% and even prevented spontaneous excitations from these cells at high concentrations of KN-93 (Vinogradova et al., 2000). Wu et al. (1999b) also showed CaMKII-mediated augmentation of LTCC current, along with evidence that inhibiting CaMKII prevented EADs resultant from slow pacing and decreased extracellular K^+^, which the authors suggest is due to CaMKII-mediated augmentation of LTCC current, leading to arrhythmia. However, the authors do not conclusively show that this effect is purely due to LTCC augmentation. Ox-CaMKII has also been shown to interact with the LTCC, which may lead to pathological consequences resultant from intracellular Ca^2+^ accumulation (Song et al., 2010). Angiotensin-II induces EADs and CaMKII activation by ROS in isolated ventricular myocytes, with augmentation of both LTCC current and late I_Na_ potentially underlying the increased propensity of EAD generation (Zhao et al., 2011). Based on the evidence for CaMKII modulating LTCC as previously discussed, this may be a key link between angiotensin-II and the augmentation of LTCC current. Diabetes may impair the functionality of the LTCC, and the impaired Ca^2+^ current of the channels has been purported to underlie the contractile dysfunction seen in a T2DM mouse cardiomyocyte model (Pereira et al., 2006). This impairment does not seem to persist in an STZ-induced T1DM rat model, with no significant difference between LTCC currents from diabetic and control animals (Al Kury et al., 2021). The role of CaMKII in regulation of LTCC during diabetes remains unclear and would benefit from further investigation.

### K^+^ Channels

Maintenance of a stable repolarization period is essential for normal cardiac function and maintenance of the cardiac action potential, as alterations in this repolarization period can lead to arrhythmia. K^+^ channels are essential for maintaining a stable resting membrane potential and termination of the AP in cardiomyocytes [for review, Ravens and Cerbai (2008)]. O-GlcNAcylated CaMKII modulates cardiac K^+^ channels through reducing transient outward K^+^ current (I_to_) and enhancing I_to_ recovery from inactivation and increased inward rectifier K^+^ current (I_K1_) (Hegyi et al., 2019; Hegyi et al., 2020), which contributes to arrhythmogenesis. Hegyi et al. (2020) further showed CaMKII is involved in downregulating expression of many of these K^+^ channels. In an acute hyperglycaemic mouse myocyte model of diabetes, Hegyi et al. (2019) showed that CaMKII and O-GlcNAcylation are necessary for the impairment seen on K^+^ channel function in DM. Contrasting this, an STZ-induced rat diabetic model showed that CaMKII was unable to regulate I_to_ inactivation in this model (Gallego et al., 2008). The differing effects of CaMKII-mediated K^+^ channel regulation in these two models highlight the issues in extrapolating results from T1 to T2DM models.

## Calcium/Calmodulin (Ca2+/CaM) Dependent Protein Kinase II, Contractility and Excitation Contraction Coupling in Diabetes

### Calcium/Calmodulin (Ca2+/CaM) Dependent Protein Kinase II and Diabetic Contractility

CaMKII also influences the impaired cardiac contractility seen in diabetes, as inhibition of the enzyme was able to restore force and rate of contraction in diabetic rat cardiac tissue (Daniels et al., 2018). Interestingly, Daniels et al. (2018) noted increased levels of O-GlcNAc modified CaMKII in human diabetic cardiac tissue. Along with findings showing that reduction of O-GlcNAc modification in the diabetic heart can improve contractile function (Hu et al., 2005), this suggests that O-GlcNAc-CaMKII may be playing an integral role in depressed diabetic cardiac contractile function. Further supporting these findings, a recent study by Hegyi et al. (2021a), Hegyi et al. (2021b) identified that O-GlcNAcylated CaMKII may be integral to the diastolic dysfunction seen in diabetes.

O-GlcNAc-CaMKII may not be the only modification of CaMKII resultant in contractile dysfunction in diabetes. Silberman et al. (2010) suggests that uncoupling of cardiac NOS mediates diastolic dysfunction. As previously discussed, NOS is known to be uncoupled in diabetes (Alp et al., 2003) leading to impaired NO production and increased levels of oxidative stress. Impairments in cardiac relaxation are a hallmark of diabetes (Fein et al., 1980; Lamberts et al., 2014; Daniels et al., 2018), so uncoupled NOS could be influencing modification of CaMKII by both nitrosylation and oxidation, contributing to the contractile dysfunction seen in diabetes.

An interaction between high glucose and oxidative stress may also be playing a role in the impaired contractility seen in the diabetic heart. Bhatt et al. (2015) observed depressed cardiac contractility resultant from high glucose conditions in T2DM ZDF rat trabeculae, which was alleviated with palmitate treatment. This palmitate treatment was able to prevent increased oxidative stress brought about by high glucose, suggesting a link between the two conditions. Considering the findings of Lu et al. (2020) that CaMKII and O-GlcNAcylation were integral to ROS production in murine ventricular myocytes, it is plausible that an interaction between high glucose, oxidative stress and CaMKII could be influencing the depressed contractility seen in DM.

### Calcium/Calmodulin (Ca2+/CaM) Dependent Protein Kinase II and Excitation Contraction Coupling in Diabetic Cardiomyopathy

CaMKII is well known to influence various processes in ECC, as highlighted in *Arrhythmogenic Targets of Calcium/Calmodulin (Ca2+/CaM) Dependent Protein Kinase II* section discussing the various channels targeted by CaMKII which may lead to arrhythmogenesis [for review, Maier and Bers (2002)]. Studies have shown impairments of ECC in DM, such as impaired contraction and relaxation in isolated T1DM rat ventricular myocytes, along with slower rates of Ca^2+^ decay (Ren and Bode, 2000). T2DM mice hearts exhibit an increased O_2_ consumption from ECC, which may be contributing to impaired cardiac efficiency (Boardman et al., 2009). These impairments affect synchrony of ECC in the cardiomyocyte, with investigators observing asynchronous Ca^2+^ release along the length of diabetic cardiomyocytes, concomitant with a reduced density of T-tubules in a T2DM mouse model (Stølen et al., 2009). Other investigators have also shown no such impairments in ECC in a T2DM rat model, despite alteration in mRNA expression of various cardiac muscle proteins (Salem et al., 2013).

It remains unclear whether CaMKII is involved in ECC impairments in diabetes, although Stølen et al. (2009) observed hyperphosphorylated CaMKII present in a T2DM mouse model, though did not link CaMKII to the ECC dysfunction. Research on the interaction between CaMKII and ECC dysfunction in diabetes remains lacking, and would prove a valuable insight into the role of CaMKII in DCM.

## Concluding Statements

There is already substantial literature demonstrating a critical role for CaMKII in various diabetic cardiac pathologies, and further research in this field continues to emerge. Thus, CaMKII presents as an appealing treatment target for diabetic cardiomyopathy, allowing the hypothetical clinician to treat many of the downstream effects of DM with one targeted therapy. A variety of different CaMKII inhibitors have already been developed with the aim to produce such a therapy [for review, Nassal et al. (2020)], however, care must be taken to ensure the physiological roles of CaMKII are not upset with such a treatment.

An isoform-specific inhibitor of CaMKIIδ (the main cardiac isoform of CaMKII) could be utilized to target the cardiac-specific pathology of autonomously activated CaMKII in diabetes, avoiding any off-target effects in other tissues, such as the *α* and *β* isoforms of CaMKII and disruption of memory formation in the hippocampus (Buard et al., 2010). Lebek et al. (2018) recently showed that the novel CaMKIIδ inhibitor GS-680 was able to reduce premature atrial contractions, DADs and EADs in human atrial tissue, showing promise for the anti-arrhythmic benefits of such an inhibitor in the clinical setting. However, this blunted arrhythmogenesis coincided with impaired contractile force in the atrial tissue (Lebek et al., 2018). This underscores the needto strike a balanceto ensure the essential physiological roles of CaMKIIδ in the heart are not compromised with such a treatment, such as its roles in both ECC and exercise training (Burgos et al., 2017; Beckendorf et al., 2018).

Considering the increased susceptibility of diabetics to arrhythmia following ischaemic injury (Chou et al., 2020), it could be of benefit to examine the effects of CaMKII inhibition in the ischaemic diabetic heart. Altering levels of CaMKII activity/expression in ischaemic diabetic myocardium could have unforeseen effects if studies focus only on the diabetic myocardium. This “hidden cardiotoxicity” has had substantial negative impacts on clinical trials and pharmaceutical therapies to date (Ferdinandy et al., 2019), so care should be taken when developing prospective therapies for the diabetic heart.

Rather than a direct targeting of CaMKII, a prospective treatment may instead look to altering the autonomous activation pathways that have become oversaturated in diabetes, preventing hyperactive CaMKII at its source. Increased O-GlcNAcylation of CaMKII may underlie various contractile abnormalities, arrhythmogenesis and even ROS generation in DM, however the modification still plays an integral role in normal physiological function. Increased O-GlcNAcylation is even protective during I/R injury (Champattanachai et al., 2007) so removing this modification may have unintended consequences on other cardiac pathologies. The dual-regulation of CaMKII by S-Nitrosylation as characterised by Erickson et al. (2015) presents a challenging task to balance the activation of CaMKII by this modification, considering the complex links between nitrosylation and both pathological and physiological processes. Oxidation has long been recognised as a pathological modification, with aberrant oxidation of proteins underlying a swathe of cardiac pathologies. A treatment targeting this modification would also present its own unique challenges, considering the complex involvement of oxidation in various intracellular signalling pathways (Zhang and Shah, 2014). Targeting any one of these modifications presents as a double-edged sword: diabetic cardiac dysfunction may be prevented by altering levels of any given PTM, but such an approach would likely have undesirable off-target effects on important physiological processes if a balance is not struck between too much and too little modification.

A recent clinical trial has examined the effects of CaMKII inhibition following anterior STEMI and is the first of its kind to examine the clinical effects of CaMKII inhibition following STEMI in human patients (Boyle et al., 2021). Despite not seeing any difference between placebo and treatment in terms of improved LV remodeling, the inhibitor appeared to be well tolerated and safe amongst the patient group. Considering the absence of adverse effects in this trial, it should hopefully pave the way for future studies to examine the effects of CaMKII inhibition in other conditions, such as DM.

Changes occurring between acute and chronic hyperglycaemia must also be considered when developing therapies for use in DM. The effects of CaMKII on cardiac K^+^ channels differ in situations of acute compared to chronic diabetes (Hegyi et al., 2020). As such, it can be assumed that blocking CaMKII activation in a patient who has had diabetes for 1 year would have noticeably different effects than in a patient who has had diabetes for 10 years. On a similar note, prediabetes presents a unique opportunity to target CaMKII before overt diabetes develops. An STZ and HFD induced rat model of prediabetes exhibited diastolic dysfunction and cardiac hypertrophy, though without increased autophosphorylation of CaMKII (Koncsos et al., 2016). Targeting CaMKII before development of diabetes could allow for a preventative treatment, minimizing the overt damage to the cardiovascular system seen in DM. However, this study did not examine the impact of other activation pathways of CaMKII in this model. O-GlcNAcylation in the rat heart is observed to be increased even before the presentation of prediabetes, following ingestion of a high fat/carbohydrate “Western” diet (Medford et al., 2012). CaMKII PTMs may be of interest in the prediabetic heart and could present a novel treatment opportunity prior to development of DCM.

Exercise has well-known beneficial effects on reducing the risk of excess cardiovascular morbidity and mortality associated with DM (Zethelius et al., 2014), however, CaMKII inhibition may blunt the benefits of exercise training. CaMKII is involved in several of the beneficial effects brought on by exercise training. Accordingly, inhibition of the enzyme prevents many (but not all) of these benefits (Kaurstad et al., 2012). Parallels can be drawn here to *β*-blocker treatment regimens and the associated exercise intolerance (Head, 1999), which can result in significant lifestyle impact for patients. Cardiac inhibition of CaMKII would again have benefits in terms of preventing pathology associated with diabetic cardiomyopathy but may negatively impact the lifestyle of prospective patients due to the resulting exercise intolerance.

As highlighted in this review, CaMKII has emerged as a key nodal signal in various pathological pathways in DM. Table 1 summarizes a search for relevant literature on the website PubMed using the terms “CaMKII,” “cardiac arrhythmia,” and “diabetes” on the 30th of May 2021. The impact of DCM on the role of CaMKII in many pathways [such as gene transcription (McKinsey, 2007)] remains to be elucidated, and could provide further treatment targets than those already described in the literature. By targeting overactive CaMKII or its downstream signalling pathways, much of the aberrant cardiovascular effects of the enzyme may be attenuated, substantially improving cardiovascular health, and minimizing the overt mortality from CVD seen in DM patients.

**TABLE 1:** Relevant literature.

First author	Journal published	Reference number	Date	Model	Arrhythmogenic events examined
Hegyi B	Circulation research	Hegyi et al. (2021a), Hegyi et al. (2021b)	2021	LV mouse cardiomyocytes (high glucose)	Ca^2+^ spark and wave frequency
				*In-vivo* ECG of STZ mice	Action Potential Duration (APD)
					DADs
					Spontaneous Action Potentials
					Arrhythmia susceptibility following Caffeine/Isoproterenol challenge
Hegyi B	Cardiovascular research	Hegyi et al. (2021a), Hegyi et al. (2021b)	2021	Atrial and ventricular cardiomyocytes from mice and rabbits (high glucose)	Na^+^ current
					Impulse propagation
					APD and STV (short term variability)
					Ca^2+^ spark and wave frequency
					DADs
Hegyi B	Basic research in Cardiology	Hegyi et al. (2020)	2020	LV cardiomyocytes (rat, rabbit and mice)	Effects on K^+^ channel current
				STZ, high fat diet and db/db mice	
Yang Y	Life Sciences	Yang et al. (2020)	2020	Electrophysiology of isolated STZ rat hearts	Conduction inhomogeneity
					Conduction velocity
					Inducible AF
Lu S	Circulation research	Lu et al. (2020)	2020	High glucose mouse ventricular cardiomyocytes	Ca^2+^ wave and spark frequency
				High glucose hiPSC-CMs	
Popescu I	Heart rhythm	Popescu et al. (2019)	2019	Surface ECG and isolated ventricular cardiomyocytes from HIP rats	Arrhythmia susceptibility following Caffeine/Dobutamine challenge
					DAD incidence
					Ca^2+^ spark frequency
Soliman H	International journal of Cardiology	Soliman et al. (2019)	2019	Isolated ventricular cardiomyocytes from STZ mice and STZ rats	Ca^2+^ transients
					SR Ca^2+^ leak
Monnerat G	Nature Communications	Monnerat et al. (2016)	2016	Ventricular cardiomyocytes, LV muscle strips and in-vivo ECG of STZ mice and STZ rats	AP prolongation
					Ca^2+^ spark frequency
					Arrhythmia susceptibility following Caffeine/Dobutamine challenge
					I_to_ current
Sommese L	International journal of Cardiology	Sommese et al. (2016)	2016	Surface ECG and isolated ventricular cardiomyocytes from fructose rich diet rats (prediabetes)	Spontaneous Ca^2+^ release events
					Spontaneous contractions
					Synchronicity of Ca^2+^ release events
					Ca^2+^ wave frequency
Rolim N	Basic research in Cardiology	Rolim et al. (2015)	2015	Ventricular intracardiac electrogram and LV cardiomyocytes from db/db mice	Inducible vent. Tachycardia
					Diastolic Ca^2+^ leak
					Increased inward current
Erickson JR	Nature	Erickson et al. (2013)	2013	Isolated HIP rat ventricular cardiomyocytes	Ca^2+^ wave and spark frequency
				Langendorff HIP rat hearts	PVCs
				*In-vivo* ECG of HIP rat hearts	Arrhythmia susceptibility following Caffeine/Dobutamine challenge
Tuncay E	Journal of bioenergetics and biomembranes	Tuncay et al. (2011)	2011	Papillary muscle strips and isolated ventricular cardiomyocytes from STZ rats	Action potential duration
